# Rapid detection of *Salmonella enterica* serotype Typhimurium in large volume samples using porous electrodes in a flow-through, enzyme-amplified immunoelectrochemical sensor

**DOI:** 10.1007/s00216-019-01901-3

**Published:** 2019-05-24

**Authors:** Joseph A. Capobianco, Joe Lee, Cheryl M. Armstrong, Andrew G. Gehring

**Affiliations:** United States Department of Agriculture, Agriculture Research Service, Eastern Regional Research Center, 600 East Mermaid Lane, Wyndmoor, PA 19038 USA

**Keywords:** Biosensor, Electrochemical sensor, Graphite felt, Immunoelectrochemistry, *Salmonella enterica*

## Abstract

Foodborne illness is a common yet preventable public health concern generating significant costs for the healthcare system, making systems to accurately detect this pathogen a topic of current research. Enzyme-based immunoassays are highly desirable because they offer shorter response times compared to traditional culture-based methods. Biosensors employing the electrochemical and optical detection of a substrate oxidized by horseradish peroxidase (HRP) have been used to successfully detect biomolecules; however, their inability to handle large sample volumes severely limits their application to food safety despite their accuracy and reliability. Here, we describe a biosensor with the capacity to process a large sample volume by utilizing an Ag/AgCl reference electrode, a platinum counter electrode, and a porous working electrode made from graphite felt coated with antibodies specific for *Salmonella* common structural antigens. This design allows samples to flow-through the electrode while capturing target pathogens. Following sample exposure, HRP-conjugated antibodies facilitate pathogen detection that culminates in an oxidation reaction with the output analyzed via Osteryoung square wave voltammetry. Detection limits of 1000 *Salmonella enterica* serotype Typhimurium cells were achieved using this newly devised flow-through, enzyme-amplified, electrochemical biosensor in samples as large as 60 mL. The low cost of the sensor allows for incorporation into disposable detection devices while its design not only broadens its applicability in sample processing but also permits the detection of various microbes by simply exchanging the antibodies.

## Introduction

*Salmonella* accounts for 42% of the total cases of bacterial foodborne illnesses and is the leading cause of foodborne hospitalization and death in the USA [1]. The Economic Research Service’s mean estimate of the annual cost of foodborne illness from *Salmonella* in 2013 was $3,666,600,031 [2], (https://www.ers.usda.gov/webdocs/DataFiles/48464/Salmonella.xlsx?v=0), [3]. Healthy People 2020 set an objective to reduce foodborne illnesses in the USA, with one of its goals being to reduce human illnesses from *Salmonella* by 25%, equating to an actual reduction from 15.0 cases per 100,000 people to 11.4 cases per 100,000 people.

Rapid and accurate identification of pathogenic bacteria is extremely important for food safety in order to prevent contaminated products from reaching the marketplace. Traditional bacterial culture enrichment technologies (i.e., broth culture and selective and/or differential plate culture) are the gold standard for detection, but it can take several days for the results to become available. Enzyme-based immunoassays are often utilized as an alternative to culture as they offer a shorter response time. In immunoassays, the antigen or antibody is labeled with an enzyme that generates a product, which is detected using optical techniques such as fluorometric, luminometric, or colorimetric detection methods.

A common enzyme-substrate reporter system is horseradish peroxidase (HRP) and 3,3′,5,5′-tetramethylbenzidine (TMB). HRP belongs to the family of heme-containing peroxidases and catalyzes the oxidation of various electron donor substrates with hydrogen peroxide. The mechanism of oxidation of the aromatic amine TMB, by peroxidase, HRP, is a well-known process [4, 5]. Oxidation of TMB by HRP/H_2_O_2_ first generates a blue-colored complex product, which turns yellow after the addition of sulfuric acid (a typically employed enzymatic “stop solution”) to the reaction medium. This yellow product has been identified as a two-electron oxidation product (diimine), which is stable in acidic solutions. It has a maximum absorbance peak at 450 nm, and it is also electroactive, thus allowing for electrochemical detection.

Biosensors utilizing electrochemical and optical detection of TMB oxidized by HRP were previously shown to be successful [6]. Although electrochemical-based biosensors are highly accurate and proven to be reliable, they characteristically cannot handle the larger sample volume associated with pathogen detection in food matrices. Separation and concentration techniques are frequently utilized in testing food samples for pathogen contamination. Although a wide variety of techniques have been reported in the literature, including centrifugation, filtration, flotation, physico-chemical adsorption, bio-specific adsorption, electrophoresis, dielectrophoresis, and liquid-liquid extraction [7], in practice most are limited to small volumes of relatively “clean” samples. The small size of bacteria (~ 1 μm) relative to eukaryotic cells (~ 10 μm) and other food particulates suggests that filtration could be very effective for rapid isolation and concentration of foodborne bacteria and has been used effectively in milk and food homogenates [8, 9].

The present study expands upon previous work [10], which utilized filtration and electrochemical detection to identify *Salmonella enterica *serotype Typhimurium*.* Here, we show that the sample volume can be dramatically increased (from 0.01 to 60 mL) through the adoption of a porous working electrode coated with antibodies specific for *Salmonella*. This design allows samples to flow-through the electrode while capturing the target pathogen. Following exposure to sample, an HRP-labeled antibody specific for *Salmonella* was added, generating a conventional sandwich assay. Ultimately, detection limits of 1000 cells were achieved using this newly devised flow-through, enzyme-amplified electrochemical sensor in both 5- and 60-mL sample volumes.

## Materials and methods

### Assay materials

The glassy carbon electrode (GCE; part # MF-2052), Ag/AgCl reference electrodes, and electrode polishing suspension were sourced from Bioanalytical Systems, Inc., (West Lafayette, IN). A 2-in.-long, 0.5-mm-diameter platinum wire counter electrode was sourced from VWR (Radnor, PA) and the 0.25-in.-thick graphite felt electrode (GFE) from Electrosynthesis (Lancaster, NY), and a second 2-in.-long, 0.5-mm-diameter platinum wire from VWR (Radnor, PA) was sourced to facilitate the electrical connection of the GFE to the electrochemical cell. Spherical borosilicate beads with a 5-mm-diameter were sourced from Thomas Scientific (Swedesboro, NJ).

Affinity purified goat anti-*Salmonella* Common Structural Antigens-Plus and horseradish peroxidase (HRP)-conjugated goat anti-*Salmonella* Common Structural Antigens-Plus antibodies along with heat-killed *Salmonella* Typhimurium cells were obtained from SeraCare (Gaithersburg, MD). For blocking solutions, Carnation nonfat powdered milk was obtained from a local supermarket, while carrageenan and bovine serum albumin (BSA) were from Sigma Aldrich (Billerica, MA). 3,3′,5,5′-tetramethylbenzidine (TMB), acetonitrile, glacial acetic acid, sulfuric acid, Tween-20, sodium acetate, and phosphate-buffered saline tablets were purchased from Sigma Aldrich (Billerica, MA). Water was deionized in-house with a Nanopure water treatment system (Barnstead, Dubuque, IA). One-Step ultra TMB ELISA substrate solution was sourced from Thermo Fisher Scientific (Waltham, MA), and 3% hydrogen peroxide was purchased from a local supermarket.

A stock solution of 0.3 mM TMB was prepared by first adding 6 mg of TMB to 4 mL of acetonitrile. The solution was then diluted with 75 mL of 0.20% sodium acetate buffer that contained 15 mL of acetonitrile and was titrated to a pH of 4.8–5.0 using acetic acid (approx. 100 μL). Prior to use, 6.3 μL of 3% hydrogen peroxide was added to the TMB solution for every 1 mL of TMB solution. The TMB solution is light-sensitive and was kept in the dark prior to use. The stop solution was prepared by diluting concentrated sulfuric acid to 1 M using Nanopure water.

Tablets were used to prepare 10-mM phosphate-buffered saline (PBS) solutions; the pH was measured to be 7.3–7.6 using a bench top pH meter (IQ Scientific). This buffer was used to reconstitute antibodies and heat-killed *Salmonella* Typhimurium cells, as well as blocking and rinsing solutions. Affinity purified goat anti-*Salmonella* Common Structural Antigens-Plus served as the primary antibody and was reconstituted to a concentration of 1 mg/mL using a 50% glycerol, 50% PBS solution. Horseradish peroxidase (HRP)-conjugated goat anti-*Salmonella* Common Structural Antigens-Plus antibody was reconstituted to a concentration of 0.1 mg/mL using a 50% glycerol, 50% PBS solution. Heat-killed *Salmonella* Typhimurium cells were reconstituted at a concentration of 10^9^ cells/mL using a 50% glycerol, 50% PBS solution. Rinsing solution was prepared by adding 0.05% Tween-20 to PBS.

### Electrode preparation

The 0.25-in.-thick GFE was cut to a 1-in.-diameter circle and soaked in 10 mL of PBS. GFE was compressed using a nitrile glove-covered hand in the PBS several times in order to wet the surface of the GFE. The GFE was then loaded into a 60-mL syringe tube from VWR (Radnor, PA) and the excess PBS was removed by depressing the syringe plunger. The GCE was polished for 30 s with two drops of 0.05 μm diamond polish on a nylon pad. The electrode was then washed with 70% ethanol to remove residual polish and then patted dry. The counter electrode was rinsed in Nanopure water prior to and after use.

### Electrode housing preparation

One small hole, approximately 0.5 mm in diameter, was drilled into the side of a 60-mL syringe tube 0.125-in. above the base of the barrel. The purpose of this hole was to provide the means for making an external electrical connection with the graphite felt. The hole was sealed from the outside using approximately 50 mg of molten wax which was from Signature Brands (Ocala, FL).

### Enzymatic product detection

Electrochemical measurements were conducted using a BAS 100B/W electrochemical analyzer (Bioanalytical Systems, Inc., West Lafayette, IN). The parameters were set for Osteryoung square wave voltammetry (OSWV) in a range of −1200 to 1200 mV with a sensitivity of 100 mA/V. Just prior to conducting the OSWV scan, the wax plug was removed and a 2-in.-long, 0.5-mm-diameter platinum wire was inserted through the hole and throughout the length of the graphite felt electrode. This wire enables the connection of the graphite felt to the BAS 100B/W electrochemical analyzer. The hole was resealed and the wire affixed using 50 mg of molten wax. Fifty borosilicate beads were added to the syringe tube in order to compress the GFE and ensure that contact conductance was maintained throughout the OSWV scan. Using the electrode holder and stand contained in the BAS 100B/W, the Ag/AgCl reference and platinum counter electrodes were inserted into the syringe tube and positioned 1.5 cm above the GFE with all electrodes connected to the corresponding alligator clip. Colorimetric measurements were made using a Tecan Safire^2^ plate reader from Männedorf, Switzerland, using an absorbance wavelength of 450 nm.

### Electrode composition comparison

**Fig. 1:**
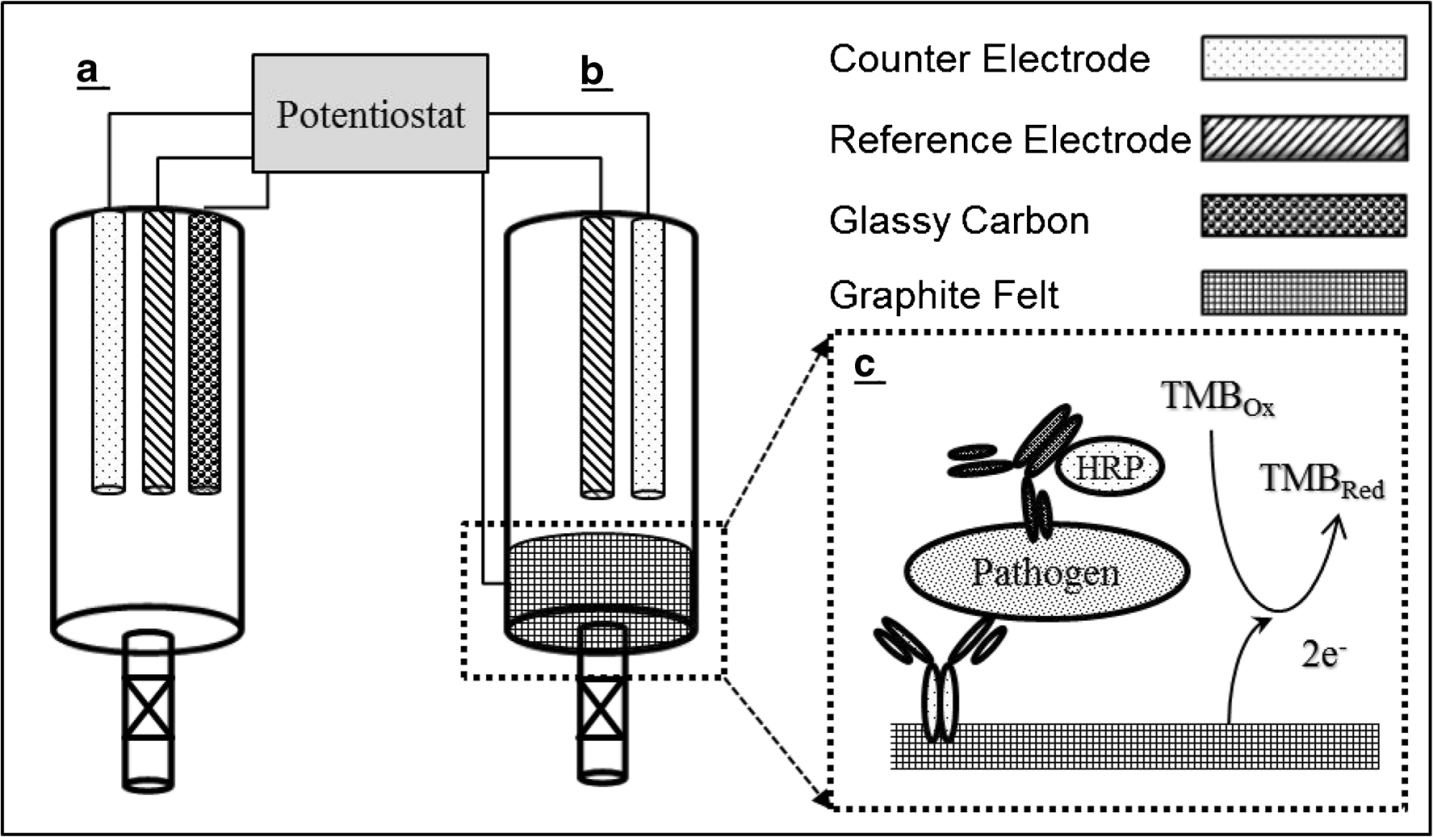
Schematic of the flow-through, enzyme-amplified immunoelectrochemical sensors. Two different materials (**a**) glassy carbon and (**b**) graphite felt were tested as working electrodes during sensor development. Both electrochemical cells utilized Ag/AgCl reference electrodes and platinum wire counter electrodes. The inset (**c**) depicts the two-site, noncompetitive immunoassay taking place at the surface of the graphite felt where the capture antibody binds the pathogen and the subsequent addition of the conjugate HRP-labeled antibody facilitates the detection of that pathogen through the oxidation of the TMB substrate

Ten-fold serial dilutions of HRP-conjugated antibody (Ab-HRP) in TMB substrate solution were prepared in a 15-mL conical tube using antibody concentrations ranging from 2.67 × 10^−9^ M to 2.67 × 10^−18^ M. The total volume of each dilution was fixed at 10 mL with a final concentration of TMB and H_2_O_2_ in each dilution of 0.3 and 5.5 mM, respectively. Following a 20-min incubation period in the dark at room temperature, 5 mL of stop solution was added. The Ab-HRP was removed from the solution using Centricon centrifuge filters from Millipore Corporation (Billerica, MA). Two hundred microliters of solution was then transferred to a 96-well plate for absorbance measurements at OD_450_. The balance of the solution, 9.8 mL, was divided equally into two 60-mL syringe tubes for the electrochemical measurements. The first tube contained the GCE (Fig. 1a), while the other utilized a graphite felt working electrode (Fig. 1b). Both tubes contained Ag/AgCl reference electrodes and platinum wire counter electrodes.

### Protein adsorption to GFE

Studies were conducted to estimate the amount of protein that can be adsorbed on the surface of the GFE. Solutions of 0.25 mg/mL of BSA in PBS were flowed through the GFE-loaded syringe tubes. Indirect UV spectroscopy measurements indicated approximately 0.482 ± 0.04 mg of BSA was the maximum quantity of protein that could be retained, which was 17.5% of the total protein that was flowed through the membrane. The approximate amount of protein that can be deposited on the GFE was determined using the following parameters: the surface area to volume ratio of the GFE, which given the porous and fibrous nature of the GFE is ~ 300 cm^2^/300 cm^3^ [11]; the physical dimensions of the membrane; the dimensions of protein sizes measured using atomic force microscopy [12]; and the assumption of hexagonal close packing of proteins on the surface. Specifically, the quantity calculated for BSA was 0.251 mg/electrode, which was in relatively close agreement to the quantity retained in the membrane through indirect measurements.

Blocking proteins were used to coat areas of the GFE not populated with antibodies in order to minimize the potential for nonspecific binding by sample or assay components to the bare GFE. Similar conditions to those used in the BSA protein adsorption studies on the GFEs were applied to the deposition of the primary antibodies, and blocking agents. Using the size of the antibody [13] and blocking proteins [14], similar estimates for the quantity of protein to coat the surface of the GFE can be calculated. Based upon physical dimensions and specific surface area of the GFE, the ratio of the number of antibodies to the number of blocking proteins was varied from 1:10 to 1:1,000,000.

### Antibody detection-fixed capture antibody

A fixed amount of capture antibody was immobilized on the surface of the graphite felt, and serial dilutions of the conjugate were used in order to simulate the capture of various pathogen cell concentrations. Rabbit anti-goat antibody from SeraCare (Gaithersburg, MD) was immobilized on the surface of the GFE followed by blocking with BSA. Theoretical calculations were conducted with the intent of depositing one antibody for every 100 BSA proteins. In order to achieve this coating, five GFEs were first immersed in 5 mL of a 4.23 × 10^−8^ M solution of rabbit anti-goat immunoglobulin G (IgG), which was then flowed through the GFEs. The eluted solution was collected, reapplied to each respective GFE, and allowed to incubate overnight. After 18–20 h, the GFE was rinsed twice with 5 mL PBST (0.5% Tween-20 in PBS) and then exposed to 6 mL of 0.25 mg/mL of BSA in PBS. The BSA solution was flowed through the GFEs with the eluted solution being collected and then reapplied to each respective GFE. The solution was then allowed to react for 30 min at room temperature. The GFEs were subsequently rinsed twice with 5 mL PBST. The positive control was not immobilized with antibody, only BSA, and for the negative control, the rabbit anti-goat IgG was substituted with anti-*E. coli* O157:H7 antibody.

Five-milliliter solutions of Ab-HRP were serially diluted from 2.7 × 10^−9^ M to 2.7 × 10^−13^ M. The dilutions were flowed through the anti-*E.coli* O157:H7 and rabbit anti-goat antibody-coated GFEs. The eluent was collected and then reapplied to each respective GFE and allowed to react for 60 min at room temperature. Following elution, the electrodes were rinsed twice with 5 mL PBST. Next, 5 mL of TMB/H_2_O_2_ solution was applied to the GFEs and allowed to react for 20 min in the dark at room temperature. Then, 5 mL of the 1-M H_2_SO_4_ stop solution was added to the container and incubated for 5 min before the electrochemical measurements were recorded. For the positive control, 0.5 mL of 2.7 × 10^−9^ M Ab-HRP containing 5 mL of 0.3 mM TMB and 32 μL of 5.5 mM H_2_O_2_ was applied, incubated for 20 min, and then processed with stop solution as above before the electrochemical measurement was conducted.

### Antibody detection-fixed antibody conjugate

The amount of capture antibody immobilized on the surface of the graphite felt was varied in order to simulate the capture of various pathogen cell concentrations. To conduct these experiments, rabbit anti-goat antibody was immobilized on the surface of the graphite felt, followed by blocking with BSA. Theoretical calculations were conducted with the intent of having ratios of Ab:BSA ranging from 1:100 to 1:1,000,000. Ten-fold serial dilutions of rabbit anti-goat IgG were prepared in order to achieve this immobilization, and five GFEs were first immersed in 5 mL of rabbit anti-goat IgG solutions ranging from 4.23 × 10^−9^ M to 4.23 × 10^−13^ M, and then flowed through the GFE. Each of the eluted solutions were collected and then reapplied to each respective GFE, and allowed to incubate overnight. The remainder of the experiment was carried out as described above for the fixed capture antibody experiments with the exception that the 5 mL solution of Ab-HRP placed on the anti-*E.coli* O157:H7 and rabbit anti-goat antibody coated GFEs was fixed at 2.7 × 10^−9^ M, instead of being serially diluted as reported in the above experiment. Again, the positive control was not immobilized with antibody, only BSA, and for the negative control, the rabbit anti-goat IgG was substituted with a 4.23 × 10^−9^ M solution of anti- *E.coli* O157:H7.

### Comparison of blocking agents

Blocking agents were compared in an effort to increase the signal to noise ratio of the assay. First, the GFE was presoaked with PBS, and then 0.25 mg/mL of BSA, nonfat powdered milk, PBST, or carrageenan (in PBS) was flowed through the respective GFE. The eluent was collected and then reapplied to each respective GFE and incubated at room temperature for 30 min. Next, each blocking solution was flowed through 5 different GFEs and subsequently rinsed with 2 × 5 mL PBST.

Five milliliters of TMB and 0.5 mL of Ab-HRP solution with a concentration of 2.7 × 10^−9^ M were reacted with the GFEs and incubated at room temperature for 20 min in the dark. Next, 5.5 mL of 1 M H_2_SO_4_ stop solution was added to the container. Five minutes following the addition of the stop solution, 400 μL of solution were removed, and the electrochemical measurements were recorded. The 400 μL sample was divided between two wells in a 96-well microtiter plate, and the absorbance was measured at 450 nm.

### Salmonella detection–capture antibody concentration

Anti-*Salmonella* antibody was immobilized on the surface of the graphite felt electrodes, followed by blocking with nonfat powdered milk. Two experimental conditions were evaluated: 1 antibody for every 10 blocking proteins compared to 1 antibody for every 100 blocking proteins. To achieve a coating of 1:10, six GFEs were first immersed in 5 mL of 4.23 × 10^−7^ M solution of anti-*Salmonella* IgG and then the solution was gravity flowed through the GFEs. The eluent was collected, reapplied to each respective GFE, and allowed to incubate overnight. This procedure was repeated for a negative control GFE where the anti-*Salmonella* IgG was substituted with anti-*E. coli* O157:H7. This same process was repeated for the 1:100 coating; however the concentration of anti-*Salmonella* was decreased to 4.23 × 10^−8^ M. Following 18–20 h, the GFE was rinsed with 2 × 5 mL PBST, and then exposed to 15 mL of 0.25 mg/mL nonfat powdered milk. The blocking solution was then flowed through the different GFEs, with the eluent being collected and then reapplied to each respective GFE. After a 30 min incubation at room temperature in the blocking solution, GFEs were subsequently rinsed with 2 × 5 mL PBST. The positive control GFE was not coated with antibody as only blocking agent was applied.

Tenfold serial dilutions of heat-killed *Salmonella* were generated to ensure that 500 to 5 × 10^7^ cells were suspended in 5-mL solutions. These cell solutions were flowed through the GFEs, with the eluted solution being subsequently returned to the vessel containing the GFE and incubated for 1 h. Following the 1-h incubation, the solution was flowed through the GFEs, and the graphite felt electrodes were rinsed 2x with 5 mL of PBST. Note, for the negative control (GFE coated with anti-*E. coli* O157:H7), this GFE was exposed to 5 × 10^7^ cells in 5 mL.

Five milliliters of Ab-HRP solutions of 2.7 × 10^−9^ M Ab-HRP was flowed through the anti-*E. coli* O157:H7 and anti-S*almonella*-coated GFEs. The eluent was collected and then reapplied to each respective GFE and incubated at room temperature for 1 h. Following elution, the GFEs were rinsed with 2 × 5 mL PBST. Next, 5 mL of a TMB/H_2_O_2_ solution was applied to the GFEs and allowed to react for 20 min in the dark. Next, 5 mL of 1-M H_2_SO_4_ stop solution was added to the container. Five minutes following the addition of the stop solution, glass beads were added to compress the GFE to ensure continuous contact with the platinum wire lead and the electrochemical measurements were recorded. The parameters for GFE measurements were OSWV mode with a scanning voltage ranging from −1200 to 1200 mV with 100-mA/V sensitivity.

One-half milliliter of 2.7 × 10^−9^ M Ab-HRP containing 5 mL of 0.3 mM TMB and 32 μL of 5.5 mM H_2_O_2_ was applied to the positive control. The solution was allowed to incubate for 20 min in the dark, and then 5.5 mL of the 1 M H_2_SO_4_ stop solution were added. Five minutes following the addition of the stop solution, the electrochemical measurement was performed as previously described.

### Salmonella detection– 60 mL sample volume

Anti-*Salmonella* antibody was immobilized on the surface of the GFE, followed by blocking with nonfat powdered milk. The 1:10 coating of six GFEs was used with the remainder of the experiment being performed as described above for the Salmonella detection-capture antibody concentration with two exceptions. First, a 60-mL volume was used instead of a 5-mL volume of 0.25 mg/mL nonfat powdered milk. Second, following the incubation of the GFEs with the heat-killed *Salmonella,* the GFEs were rinsed with 60 mL of PBST instead of the 5 mL used in the lower-volume experiments. Upon rinsing the electrode, the same protocol employed for 5 mL samples was utilized for incubation of the electrode with the chemical substrate and electrochemical detection of the enzymatic product. Further, the positive control GFE was not coated with antibody as it was only blocked as stated above and for the GFE reacted with anti-*E.coli* O157:H7, this felt was exposed to 5 × 10^7^ cells in 5 mL.

## Results and discussion

### Electrode composition

**Fig. 2:**
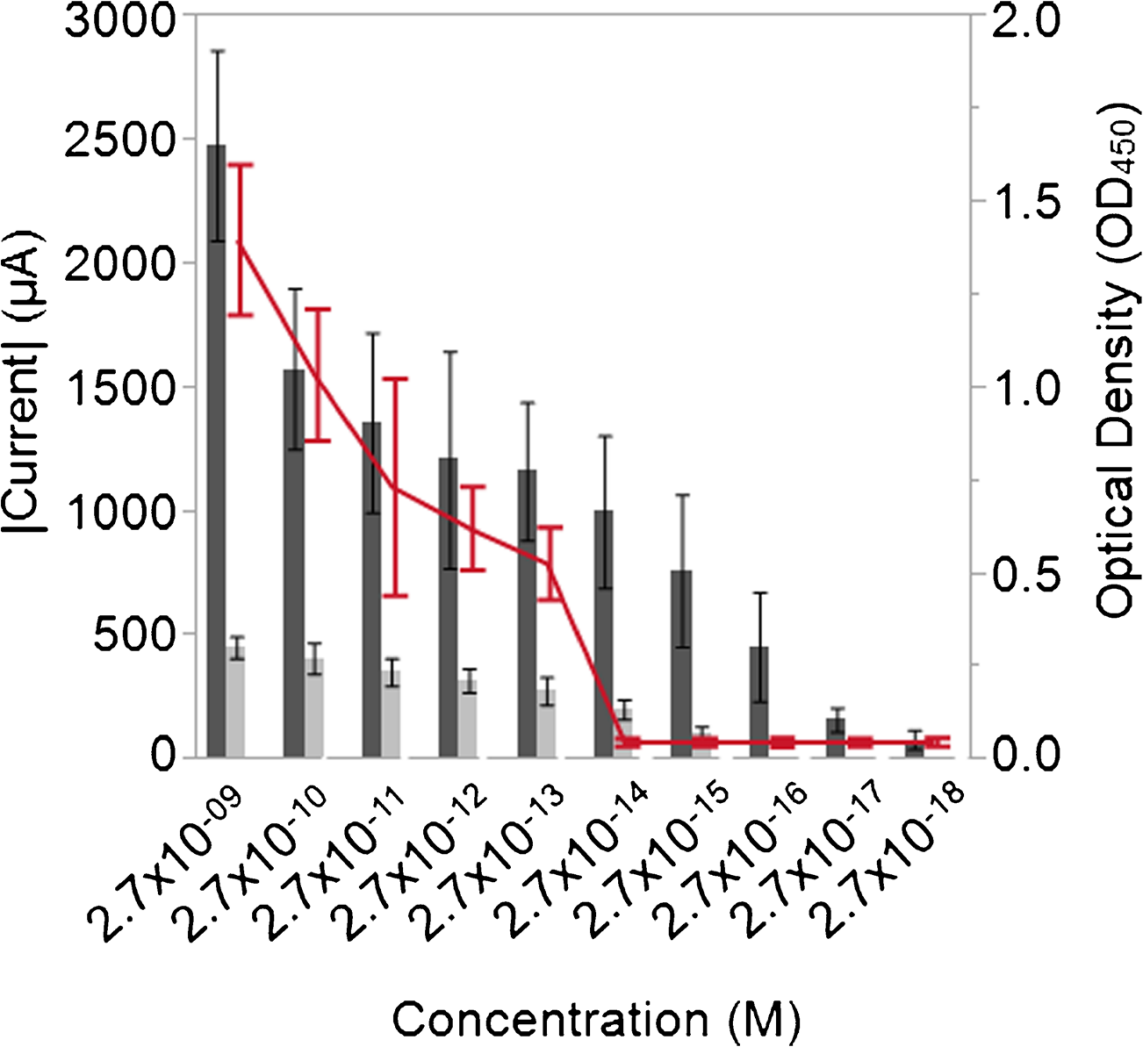
Graphite felt electrodes display higher signal intensities compared to glassy carbon. The effect of the electrode composition was determined by measuring the response of the graphite felt (dark gray bars) and glassy carbon (light gray bars) to various concentrations of Ab-HRP. The absolute value of the current (y-axis on the left measured) at −340 mV for both the graphite felt and GCEs is reported in addition to the optical density reading (red line) of the Ab-HRP dilutions (y-axis on the right). Measurements represent the average values of three independent experiments with error bars indicating the standard error of the mean

The effect of using different materials for the working electrode was determined by measuring the response of graphite felt and glassy carbon to various concentrations of Ab-HRP (Fig. 2). The optical densities of the reactions were also measured to verify that the enzymatic reaction proceeded as expected. The y-axis on the left side of the graph denotes measurements of electric current at −340 mV in microamps (μA) for both the graphite felt and GCEs while the y-axis on the right reports the optical density reading of the Ab-HRP dilutions with the TMB substrate and stop solution added. Note that because electric current values were negative, the absolute value of the measurement was utilized in order to graphically represent the results of both the optical and electrochemical measurements.

From Fig. 2, both the current and OD measurements demonstrate an apparent dose response associated with the concentration of the conjugate. Using an estimated molecular weight of 370,000 g/mol (using the vendor specification of ~5 HRP molecules per antibody with a MW of 44,000 g/mol for HRP and a MW of 150,000 g/mol for IgG) for the HRP-conjugated antibody, the limit of detection (LOD) for the GCE was calculated to be 2.7 × 10^−15^ M, which is fairly consistent with 8.5 × 10^−14^ M [15] and 2.2 × 10^−14^ M [6] values that have been reported in the literature for carbon electrodes. The LOD for the graphite felt electrode was 2.7 × 10^−17^ M, which was two logs more sensitive than the GCE. Several factors may have contributed to the lower LOD for the graphite felt electrode, including having a larger surface contact area, possibly an improved ability to conduct an electrical charge, and/or the fact that the diffusion distance is smaller compared to the GCE. In addition to its increased sensitivity, the graphite felt electrode is believed to be the superior material for the construction of a working electrode because it can be purchased at a much lower cost than the GCE.

### Antibody detection

**Fig. 3:**
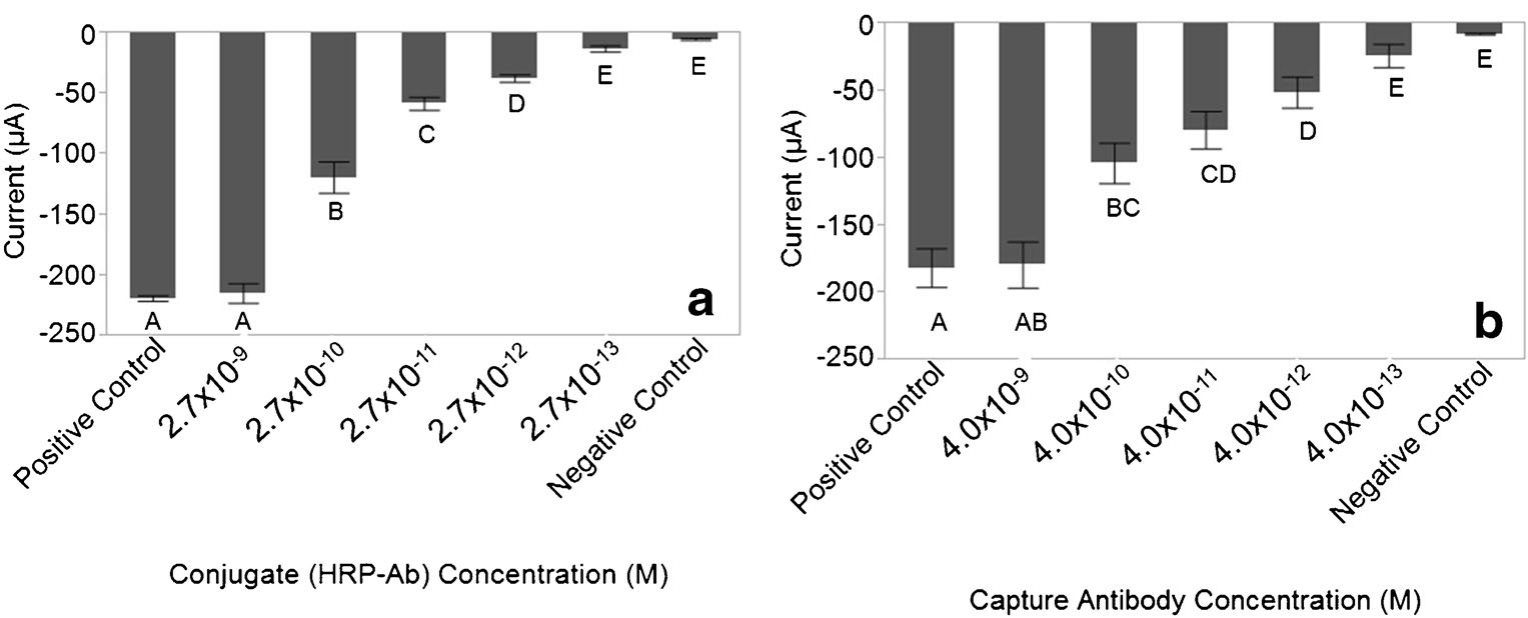
Detection capacity of the graphite felt electrode. (**a**) Current response generated by the graphite felt electrode using a fixed concentration of capture antibody and various levels of conjugate (10^−9^ to 10^−13^). (**b**) Current response generated by various amounts of capture antibody (10^−9^ to 10^−13^) and a fixed concentration of conjugate. Bars represent the average current response generated for each of the four trials with the error bars representing one standard deviation of the mean. Significance between measured electric current signals is noted by different letters as determined by Student’s t test at a 95% confidence level

To assess the capability of the graphite felt to function as a working electrode in a sensing platform, we analyzed its ability to detect conjugate using a rabbit anti-goat antibody as a capture antibody for the HRP-labeled goat anti-*Salmonella* conjugate (Fig. 3). The detection of the conjugate was performed under two experimental conditions. The first utilized a fixed amount of capture antibody on the surface of the felt with serial dilutions of conjugate solution (Fig. 3a), and the second used varying amounts of capture antibody with a fixed concentration of conjugate (Fig. 3b) to simulate the capture of different concentrations of *Salmonella* cells.

Upon fixing the capture antibody concentration, solutions containing serial dilutions of the conjugate were assayed (Fig. 3a). The current responses for each level of conjugate concentration were compared using Student’s t test with α = 0.05. Student’s t could not differentiate the positive control from 2.7 × 10^−9^ M (*p* = 0.643) nor could the negative control be differentiated from 2.7 × 10^−13^ M (*p* = 0.3976). However, with the exception of 2.7 × 10^−13^ M, individual Student’s t tests indicate that the data generated for each concentration was statistically different from one another for every tenfold dilution (*p* < 0.0001). Based on these data, the sensor has a dynamic range of four orders of magnitude and displays sufficient resolution to provide a quantitative response. From the individual t tests, the smallest discernable concentration of conjugate that was statistically different from the negative control was 2.7 × 10^−12^ M (*p* = 0.0019).

For the next set of experimental conditions, the amount of capture antibody on the electrode was varied while the concentration of conjugate was fixed (Fig. 3b). This experiment more closely simulates the response of a sandwich assay, given that the conjugate concentration was fixed while the amount of capture antibody was varied. The current responses for each level of conjugate concentration were compared using Student’s t test with α = 0.05. Student’s t test could not differentiate the positive control from 4.2 × 10^−9^ M (*p* = 0.9059), nor could the negative control be differentiated from 4.2 × 10^−13^ M (*p* = 0.3434). Similar to the other antibody detection experiment, this assay also displays a dose response and dynamic range of approximately four orders of magnitude. Individual Student’s t tests indicate that the data generated for each concentration was statistically different from one another for every 100-fold dilution (*p* < 0.0044). While the assay cannot distinguish signals with the tenfold resolution observed in the data presented in Fig. 3a, the test can still be quantitative. From the individual t tests, the smallest concentration of conjugate that can be discerned to be statistically different from the negative control is 4.2 × 10^−12^ M (*p* = 0.0160), which was on the same order of magnitude as that observed when the fixed capture antibody concentration was fixed.

There was approximately a tenfold reduction in signal amplitude observed when the measured currents in the electrode comparison experiments were compared with the antibody detection experiments (Fig. 3). The major difference between these experiments was that in the electrode comparison experiment (Fig. 2), the GFE was bare and the conjugate was removed from the solution prior to exposure to the electrode, while the antibody detection experiments utilized protein coated electrodes (Fig. 3). The antibodies on the graphite felt electrode were necessary to provide selectivity, while the targeted function of BSA was to prevent nonspecific binding. To determine if the blocking agent had an impact on the intensity of the signal, several different blocking agents were tested, while retaining the same ratios of antibody and blocking agent on each electrode. Antibody coating without a blocking agent was also evaluated under antibody coverage conditions consistent with the other electrodes.

**Fig. 4:**
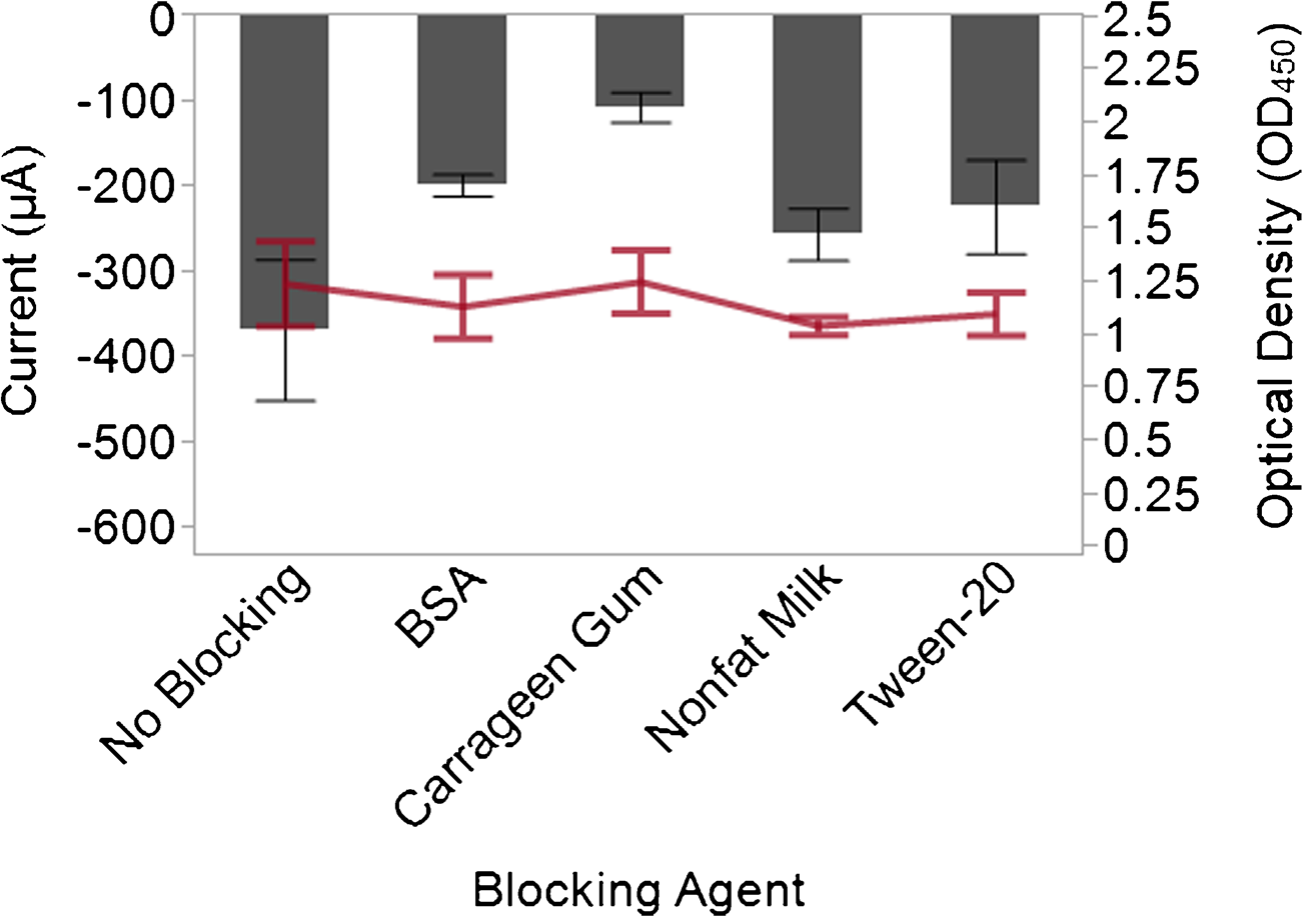
Blocking agents can affect the amplitude of the signal generated by the working electrode. The current (y-axis on the left) generated by the graphite felt at −340 mV when BSA, carrageenan gum, nonfat milk, and Tween-20 were used as blocking agents was measured (dark gray bars). The absence of a blocking solution was also tested as a control. In addition, the optical density readings (red horizontal line) of the solutions were recorded (y-axis on the right). Measurements represent the average values of three independent experiments with error bars indicating the standard error of the mean

Figure 4 depicts the measured current for different blocking agents along with the measured optical density of the solution. The OD responses for each blocking agent were analyzed using an ANOVA. These analyses indicate that it is unlikely that the measured OD responses were affected by the blocking agent employed because the *p* value was 0.788. Although the blocking agent on the graphite felt electrode does not appear to have an effect on the OD measurements, it does appear to have an effect on the measured current as the p value for the ANOVA was calculated to be 0.0115. The blocking agent utilized can affect the amplitude of the current measured at −340 mV. Relative to antibody-coated electrodes without a blocking agent, antibody-coated electrodes blocked with BSA, carrageenan gum, and Tween-20 displayed smaller response currents. However, the current response for the antibody-coated electrode blocked with nonfat milk was not statistically distinct from the antibody-coated electrode without a blocking agent. Since the nonfat milk displayed the least impact on the measured response, it was selected as the blocking agent in subsequent experiments.

**Fig. 5:**
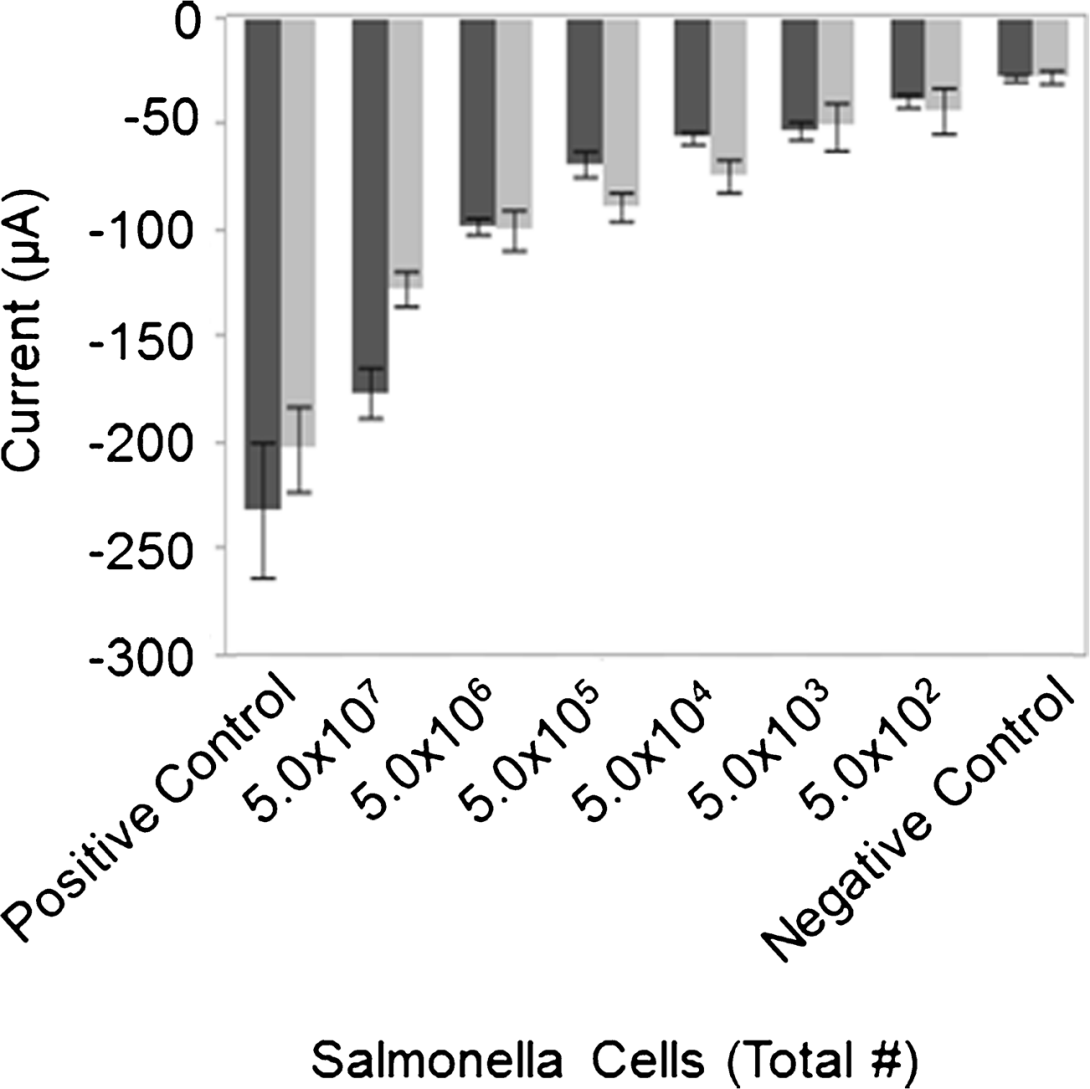
Detection of *Salmonella enterica* serotype Typhimurium using a porous electrode in a flow-through, enzyme-amplified immunoelectrochemical sensor. The current at −340 mV was measured upon exposure of the sensor to various concentrations of *Salmonella* cells ranging from 10^2^ to 10^7^. Cells were suspended in two different sample volumes with the response of each sample volume indicated (dark gray bars representing 5 mL and light gray bars representing 60 mL). Measurements represent the average values of three independent experiments with error bars indicating the standard error of the mean

To determine if the sensor could be applied to detect foodborne pathogens, the response of the sensor to a suspension containing *Salmonella* cells was measured (Fig. 5). Two different suspension volumes (5 and 60 mL) containing an identical number of heat-treated *Salmonella enterica* serotype Typhimurium cells were tested. This experiment was replicated three times for each volume. Qualitatively, one can see a dose-dependent response of the sensor that correlates to the number of cells in the test suspension, and that response appears to be independent of sample volume. However, upon closer examination of the data, it becomes apparent that there was a larger variation between trials in the 60 mL data, which affects the lower limit of detection. When the sample volume is 5 mL the electric current signal for 5000 cells can be differentiated from the negative control (*p* = 0.0089) while the signal for 500 cells cannot be differentiated from the negative control (*p* = 0.2106). When the data for the 60-mL volume was analyzed for 50,000 cells it can be differentiated from the negative control (*p* = 0.0017) while the signal for 5000 cells (*p* = 0.0739) and 500 cells (*p* = 0.2066) cannot be differentiated from the negative control. The standard deviation in the data for the 60-mL volumes is higher for each level than that for the 5-mL volume and prevents the 5000 cell sample from being statistically different from the negative control, thus making the limit of detection higher when the larger volume of liquid was used.

## Conclusion

In conclusion, the porous electrode flow-through immunoelectrochemical sensor presented here significantly expands the applicability of this technology from the past work reported by this research group [10]. Several of the improvements were replacement of a gold mesh working electrode with relatively inexpensive graphite felt, a large increase in sample size (1 to 60 mL), improvement of signal to noise ratio via reduction of nonspecific binding by enzyme-antibody conjugate, an expanded dynamic range of detection concomitant with a large working electrode surface area, and elimination of a pump-driven system in favor of a gravity-flow design. The previous automated flow-through immunoelectrochemical detection system had an apparent detection limit of approx. 10^5^*E. coli* O157:H7 cells/mL in sample sizes of approx. 0.1 to 1 mL [10]. In comparison to these results, the flow-through immunoelectrochemical assay investigated in this work was demonstrated to have an increased sample size by a factor of 60× to 600× and a conservatively calculated limit of detection reduced by approx. two orders of magnitude; 833 cells/mL (60-mL sample volume) 1000 cells/mL (5-mL sample volume). In another comparable immunoelectrochemical assay that employed an antibody-coated filter membrane proximal to a hollow carbon rod working electrode, results indicated detection of *Salmonella* to be approx. 100 cells/mL with a 1-mL sample volume [16].

Given that the sample volume is seemingly unlimited for this detection platform and that the flow-through design may permit sample volumes to be greatly increased beyond what was tested here, it may be possible with this newly devised biosensor to broaden testing applications to large-volume samples such as rinsates, wash waters, or irrigation waters. In addition, although we used this biosensor to detect *Salmonella enterica*, this type of sensor could be used to detect a variety of pathogens or microorganisms in general by simply exchanging the antibodies on the graphite felt. Lastly, the low cost of the graphite felt would ultimately allow the design to be incorporated into a disposable detection device.
